# Eco-friendly Dielectric Insulation Materials with Life Cycle Sustainability

**DOI:** 10.34133/research.0986

**Published:** 2025-11-06

**Authors:** Jun-Wei Zha, Wenye Zhang, Yang Zhang, Baoquan Wan

**Affiliations:** ^1^ State Key Laboratory of Alternate Electrical Power System with Renewable Energy Sources, School of Electrical and Electronic Engineering, North China Electric Power University, Beijing 102206, P. R. China.; ^2^ School of Chemistry and Biological Engineering, University of Science and Technology Beijing, Beijing 100083, P. R. China.; ^3^ State Key Laboratory of Power System Operation and Control, Department of Electrical Engineering, Tsinghua University, Beijing 100084, P. R. China.

## Abstract

Modern electrical infrastructure relies heavily on insulation materials. Critical examples include polyimide films in microelectronics, epoxy resins in transformers, silicone rubber on outdoor insulators, and polyolefins in power cables. These solid insulation materials have high resistivity, which provides reliable assurance of the safe operation of equipment and power grids. However, both their production and end-of-life disposal place an important environmental burden. In light of the growing prevalence of sustainability principles and circular economy concepts, researchers and industry experts are re-evaluating every stage of the insulation life cycle. This includes synthesis processes, raw materials, energy consumption, and generation of toxic by-products, alongside performance durability and health impacts during use. Ultimately, disposal or recycling methods must also be considered.

## Adopting a Life Cycle Perspective

**Fig. 1.**
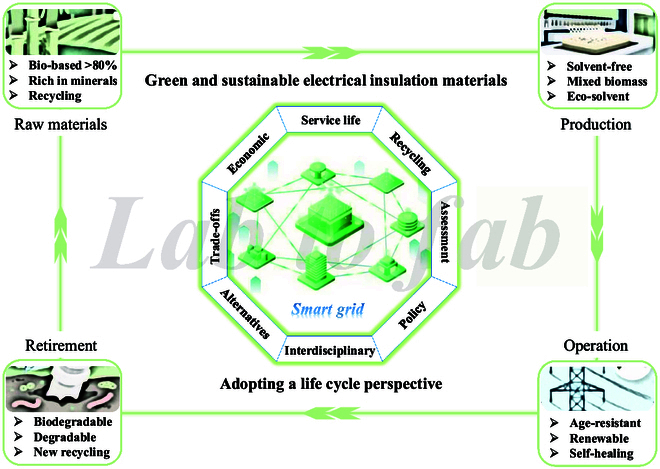
Conceptual diagram of eco-friendly dielectric design throughout the entire life cycle.

Sustainability in polymer insulation demands considering impacts at each phase (Fig. 1). Raw material sourcing is pivotal. Conventional petrochemical monomers contribute to greenhouse emissions and resource depletion, whereas bio-based feedstocks or abundant minerals (for silicones) can reduce the carbon footprint [1]. For instance, producing polyethylene from renewable sugarcane ethanol can remove more CO_2_ than it emits [2]. Manufacturing processes also matter. Polymerization routes often involve hazardous solvents or catalysts. For example, polyimide precursor resins traditionally use polar aprotic solvents like *N*-methyl-2-pyrrolidone, which poses health and environmental risks [3]. Replacing such chemicals with greener alternatives or solvent-free routes improves the sustainability profile. During the usage phase, high-performance insulation materials can improve the service life of equipment by healing defects during operation to achieve sustainability. However, the optimization and simplification of healing conditions seem to be the key to material design [4]. Finally, for insulators based on thermosetting materials, the end-of-life stage can be considered their fatal weakness. Thermally cross-linked polyimides, epoxy resins, and silicone rubbers do not melt and are difficult to recycle. If landfilled, they can persist for centuries. Incineration can recover energy but may release harmful compounds, especially halogenated polymers. The ideal vision is a circular economy for insulation materials, where polymers are either recyclable into new products or biodegradable/compostable after fulfilling their purpose. The core challenge of recent research efforts is to achieve this goal without compromising strict electrical and thermal performance [5].

Among all material categories, some cross-disciplinary design strategies are emerging. One of them is to use monomers from renewable/biological sources to replace petrochemical precursors while achieving comparable polymer properties. Another strategy is to introduce dynamic or degradable bonds into polymer networks to achieve healing, reprocessing, or chemical recycling of thermosetting materials. The third strategy is to eliminate harmful substances from the formula, for example, shifting toward halogen-free flame retardants and solvent-free processing, to reduce the environmental impact during the production process and the impact in case of fire or disposal. Importantly, these innovations must transition from laboratory-scale feasibility to industrial-scale manufacturing (lab to fab) to have a real impact.

## Lab to Fab

In recent years, a major technological advance has been the move toward thermoplastic high-voltage (HV) cable insulation. Researchers and industry leaders have demonstrated that polypropylene (PP)-based compounds formulated for this purpose can replace cross-linked polyethylene in medium-voltage and HV cables, offering equivalent electrical performance and being fully recyclable. Several groups achieved PP-based insulations that pass the stringent electrical tests for HV cables [6,7]. This new type of cable insulation material avoids peroxide cross-linking and has a faster processing speed. This not only saves energy and time during production, but this also means that when the cable reaches the end of its service life, its polymers can be melted down and reprocessed. The alliance also emphasizes that even if recycled PP is downgraded and processed into other products, it is feasible, which helps achieve the goal of circular economy.

Now, with better materials science and the imperative of sustainability, the lab-to-fab transition is finally happening for thermoplastic HV cables. The move from cross-linked polyethylene to thermoplastic PP insulation in HV cables is a flagship example of translating lab innovation into an industrial solution that checks all sustainability boxes. This shows that even established sectors such as the power cable industry can evolve in response to environmental concerns. Remaining challenges include ensuring that new materials meet all reliability requirements and establishing the logistical framework to reclaim and recycle materials after 30 to 40 years of use. However, given the progress made in recent years, it is optimistic to believe that polyolefins will increasingly become part of a closed-loop economy for electrical infrastructure. This demonstrates the proactive transition of traditional industries toward a green circular economy. Achieving this sustainable objective requires interdisciplinary collaborative innovation as its foundation.

## Future Directions and Perspectives

Providing green and sustainable insulation materials is a multidisciplinary challenge that requires innovation in chemistry, materials science, and industrial engineering, guided by environmental science and policy. We will outline key future directions and considerations for achieving sustainable advanced dielectrics.

Service life: The development of next-generation sustainable insulation materials will consider their lifespan from the outset. This means selecting polymer chemical compositions that can be depolymerized or reprocessed. Where feasible, reversible bonds should be incorporated, including copolymers and biodegradable linkers. At the same time, any factors that hinder recycling should be avoided, such as toxic additives or excessive compounds. For example, future epoxy or polyimide systems may utilize dynamic covalent bonds (reversible chemical bonds that allow reprocessing) to achieve self-healing and recyclability, as demonstrated at the laboratory scale [8,9]. Similarly, thermoplastic solutions are preferable to thermosetting solutions where performance permits. Modular design concepts can also be beneficial. If an insulation component can be easily separated from the rest of the device, it is easier to replace or recycle without discarding the entire component.

Recycling technologies: Continuous research and development of recycling processes specifically targeting insulation polymers is of critical importance. This includes catalytic pyrolysis for silicones and cross-linked hydrocarbons, solvent-based decomposition methods for thermosetting materials, and improved mechanical recycling for thermoplastic materials (efficient sorting, cleaning, and re-extrusion, as well as performance restoration). However, catalytic pyrolysis has high energy consumption and investment, but its processing capacity is large and scalable. The solvent method has mild conditions and low energy consumption, but the cost and recovery of solvents limit its large-scale application. Each cost-effectiveness needs to be optimized. Furthermore, the development of selective depolymerization technologies is of great interest, such as chemicals that can break down specific polymer types under moderate conditions. Similarly, biological recycling, such as enzymes or microorganisms that can digest polymers, is also an area of focus. In the field of chemical reuse, such as converting polymer waste into higher-value materials, there may also be potential applications [10,11].

Life cycle: The development of sustainable materials must be guided by quantitative life cycle assessments to avoid the pitfalls of shifting burdens. For example, a bio-based monomer that requires a very complex processing procedure may generate more emissions than the petrochemical product it replaces. Similarly, adding a recycling step that consumes large amounts of energy or harmful chemicals may offset the environmental benefits. Therefore, it is extremely important to focus on life cycle assessments in the early stages of insulation material research and development. Moreover, eco-friendly insulation materials exhibit electrical properties comparable to those of their traditional counterparts, although their heat resistance is typically inferior and their mechanical strength may be slightly weaker. This shortcoming necessitates compensation through the incorporation of fillers or modification techniques.

Alternatives: In some cases, sustainability may also mean using alternative insulation materials. For example, there is cellulose-based insulation paper for transformers, as well as lignocellulosic paper for purification [12,13]. These materials are bio-based and have established recycling (or biodegradation) pathways. Improvements can also be made to impregnating agents, such as biodegradable natural ester oils. This novel natural ester oil is eco-friendly, boasts a long service life, and exhibits outstanding high-temperature resistance. Even HV insulation systems can be primarily composed of renewable, biodegradable components. Perhaps for a specific application, the optimal sustainable solution is not to tweak existing polymers but to completely replace them with a different insulation material. For example, the use of sulfur hexafluoride (SF_6_) gas in switchgear has prompted the search for alternatives to vacuum or solid insulation materials [14].

Interdisciplinary: The search for sustainable insulation materials requires close collaboration between electrical engineers, chemists, and environmental scientists. Electrical engineers set performance targets and test protocols. Chemists and materials scientists design formulations to meet these targets. Environmental scientists ensure that these formulations achieve the goal of reducing impact. They work together, continuously improving, to find the best solutions.

Performance and safety: Not every green material is superior in every aspect. Durable insulation materials help improve the safety and reliability of power systems [15–19]. Any new sustainable material must meet or exceed the performance levels of existing materials to be viable. Early adoption is likely to focus on areas where sustainability can be achieved without compromise. For example, recyclable PP cables not only offer recyclability but also accelerate production speeds and may have higher current ratings. Such a win-win situation will drive initial success. In more challenging areas, replacing traditional materials with the goal of meeting stringent requirements may require more time.

Policy and producer: The regulatory framework will have an important impact on the sustainability of materials. Certain bans and restrictions are gradually phasing out some chemicals. For example, materials based on bisphenol A in food environments, specific siloxanes (D4, octamethylcyclotetrasiloxane) in consumer products, and brominated flame retardants in electronic devices. These regulations often promote innovation by forcing the search for alternatives. In the field of electrical insulation, extended producer responsibility laws may require manufacturers or utilities to recycle insulation materials at the end of a product’s lifespan. Standards and procurement policies can also play a role. If green public procurement guidelines stipulate that new infrastructure must use recyclable or bio-based insulation materials with a certain content, this would create market pull. Industry alliances may also establish voluntary standards for “eco-friendly insulation materials”, specifying criteria for their composition and recyclability. Another policy aspect involves incentives for using recycled materials. For example, tax credits or subsidies could be provided for cables containing recycled polymer components. On the other hand, waste bans, such as prohibiting the landfilling of large composite structures, would encourage the establishment of recycling channels. The policies can both provide incentives and set restrictions to accelerate the adoption of sustainable materials.

Economic: Finally, for sustainable insulation materials to flourish, they must be economically attractive. This is becoming increasingly apparent as environmental costs are factored into the equation. The need to pay disposal fees or regulatory fines for nonrecycled materials may promote the development of recyclable materials. Similarly, if a carbon tax is implemented, bio-based polymers may gain a cost advantage. Additionally, becoming a leader in sustainable technology can attract investors and customers who prioritize environmental, social, and governance factors. Circuit boards and components that are halogen-free and contain recycled materials may attract more investor attention. Although the electrical insulation industry has traditionally been conservative, it is inevitably influenced by these trends.

The field of electrical insulation materials is on the verge of a green transformation. Representative success stories such as recyclable wind turbine blades and thermoplastic recyclable cables demonstrate that sustainable high-performance insulation materials are not only feasible but are also being realized [20]. The journey from laboratory discovery to full-scale industrial application is fraught with challenges, but the progress made to date gives us reason for optimism. By continuing to integrate green chemistry, leveraging interdisciplinary collaboration, and aligning with forward-thinking policies, researchers and engineers can provide sustainable insulation materials for the electrical sector.

## References

[1] Li JL, Liu M, Liu Y, Zhao P, Lou YH, Meng ZQ, Song XX, Hu ZL, Liu YZ, Yu HP. Processable bio-based polybenzoxazine with tunable toughness and dielectric properties. Research. 2025;8:0745. 40556943 10.34133/research.0745 PMC12187022

[2] Jiang Y, Li J, Li D, Ma YK, Zhou SC, Wang Y, Zhang DH. Bio-based hyperbranched epoxy resins: Synthesis and recycling. Chem Soc Rev. 2024;53:624–655. 38109059 10.1039/d3cs00713h

[3] Dong XD, Wan BQ, Zha JW. Versatile landscape of low-*k* polyimide: Theories, synthesis, synergistic properties, and industrial integration. Chem Rev. 2024;124(12):7674–7711. 38847509 10.1021/acs.chemrev.3c00802

[4] Wan BQ, Zha JW, Dang ZM. Chemical structure design for eco-friendly dielectric polymer materials. Prog Polym Sci. 2025;169: Article 102014.

[5] Wan BQ, Xiao MY, Dong XD, Yang X, Zheng MS, Dang ZM, Chen G, Zha JW. Dynamic covalent adaptable polyimide hybrid dielectric films with superior recyclability. Adv Mater. 2024;36(52):2304175. 10.1002/adma.202304175 37382198

[6] Zhou Y, Dang B, Wang HM, Liu JP, Li Q, Hua J, He JL. Polypropylene-based ternary nanocomposites for recyclable high-voltage direct-current cable insulation. Compos Sci Technol. 2018;165:168–174.

[7] Li JY, Yang K, Wu KN, Jing ZH, Dong JY. Eco-friendly polypropylene power cable insulation: Present status and perspective. IET Nanodielectr. 2023;6(3):130–146.

[8] Sun WJ, Xu JZ, Song JH, Chen Y, Lv ZP, Cheng YH, Zhang L. Self-healing of electrical damage in insulating robust epoxy containing dynamic fluorine-substituted carbamate bonds for green dielectrics. Mater Horiz. 2023;10:2542–2553. 37070696 10.1039/d3mh00040k

[9] Wan BQ, Yang X, Dong XD, Zheng MS, Zhao QL, Zhang HK, Chen G, Zha JW. Dynamic sustainable polyimide film combining hardness with softness via a “*Mimosa*-like” bionic strategy. Adv Mater. 2023;35(2):2207451. 10.1002/adma.202207451 36281805

[10] Fan LX, Chen L, Zhang HY, Xu WH, Wang XL, Xu SM, Wang YZ. Dual photo-responsive diphenylacetylene enables PET in-situ upcycling with reverse enhanced UV-resistance and strength. Angew Chem Int Ed Engl. 2023;62(52): Article e202314448. 37938175 10.1002/anie.202314448

[11] Wong WL, Xu JH, Zhao Y, Wang YD, Du H, Zhang JH, Kang YQ, Chen YQ, Kang FY, Li BH. Upcycling of degraded Prussian blue into layered materials for sodium-ion battery. Research. 2025;8:0643. 40123995 10.34133/research.0643 PMC11927955

[12] Adekunle AA, Oparanti SO, Fofana I. Performance assessment of cellulose paper impregnated in nanofluid for power transformer insulation application: A review. Energies. 2023;16(4):2002.

[13] Wang B, Wang JM, Hu ZH, Zhu AL, Shen XJ, Cao XF, Wen JL, Yuan TQ. Harnessing renewable lignocellulosic potential for sustainable wastewater purification. Research. 2024;7:0347. 38576863 10.34133/research.0347 PMC10993153

[14] Hu SZ, Wang Y, Zhou WJ, Qiu R, Luo YB, Wang BS. Dielectric properties of CF_3_SO_2_F/N_2_ and CF_3_SO_2_F/CO_2_ mixtures as a substitute to SF_6_. Ind Eng Chem Res. 2020;59(35):15796–15804.

[15] Zhang YL, Ruan KP, Zhou K, Gu JW. Controlled distributed Ti_3_C_2_T*_x_* hollow microspheres on thermally conductive polyimide composite films for excellent electromagnetic interference shielding. Adv Mater. 2023;35(16):2211642. 10.1002/adma.202211642 36703618

[16] Yang X, Ren JW, Wan BQ, Qin SC, Wang Q, Huang WJ, Gao JH, Xia B, Zha JW. High toughness, healable, self-cleaning polydimethylsiloxane elastomers with “rigid-while-flexible” mutual network structure. Mater Horiz. 2024;11:5058–5069. 39102285 10.1039/d4mh00409d

[17] Xiang PC, Wan BQ, Huang WJ, Yang X, Yang XY, Xia B, Jung YC, Zha JW. Synergistic dual effect dynamic network regulation of self-healing silicone rubber composites for outdoor insulation. Compos Commun‌. 2025;56: Article 102418.

[18] Yang X, Huang WJ, Hao D, Zha JW. Smart polydimethylsiloxane materials: Versatility for electrical and electronic devices applications. Adv Mater. 2025;37(17):2500472. 10.1002/adma.202500472 40091339

[19] Chen J, Shen ZH, Kang Q, Qian XS, Li ST, Jiang PK, Huang XY. Chemical adsorption on 2D dielectric nanosheets for matrix free nanocomposites with ultrahigh electrical energy storage. Sci Bull. 2022;67(6):609–618. 10.1016/j.scib.2021.10.011 36546122

[20] Clarke RW, Rognerud EG, Puente-Urbina A, Barnes D, Murdy P, McGraw ML, Newkirk JM, Beach R, Wrubel JA, Hamernik LJ, et al. Manufacture and testing of biomass-derivable thermosets for wind blade recycling. Science. 2024;385:854–860. 39172828 10.1126/science.adp5395

